# Patient Identification of the most important outcomes in a gastrointestinal behavioral health program

**DOI:** 10.1186/s41687-026-01125-x

**Published:** 2026-07-06

**Authors:** Robert E. Brady, Brooke A. Duarte, Corey A. Siegel, Jessica K. Salwen-Deremer

**Affiliations:** Dartmouth–Hitchcock Medical Center, Lebanon, USA

**Keywords:** Patient-reported outcomes, Psychogastroenterology, Behavioral health, Disorders of gut-brain interaction, Inflammatory bowel disease

## Abstract

**Background:**

Brain-gut behavior therapies (BGBTs) are evidence-based treatments for patients with disorders of gut-brain interaction (DGBI) and inflammatory bowel disease (IBD). While BGBT clinical trials prioritize decreasing symptom severity and improving anxiety, depression, and quality of life, these outcomes are not designed based on patients’ priorities. We aimed to determine the optimal patient-centered outcome measures to assess change following BGBT using a stakeholder-engaged qualitative process.

**Methods:**

We interviewed patients 1:1 with DGBI and IBD with and without BGBT experience. Interview data were analyzed using rapid qualitative analysis. Subsequent focus groups reviewed the qualitative findings to verify accuracy, identify high-priority and missing constructs, and create clinical use recommendations. Measure recommendations were developed based on patients’ perspectives and the extant literature.

**Results:**

Qualitative interviews (*N* = 20) yielded five domains: lived experience of disease, how to cope with illness, perceived value of behavioral intervention, outcomes and measurement, and behavioral intervention effects. Focus group participants (*N* = 14) broadly confirmed that the qualitative themes represented their experience with BGBTs and prioritized confidence in symptom management, social connectedness, lived experience with disease, and mental/behavioral health symptoms. They highlighted the importance of brevity, semi-regular re-evaluation, and access to results. Resultant measures included: Brief Illness Perception Questionnaire, PROMIS Self-Efficacy for Managing Chronic Conditions-Symptoms, UCLA Loneliness Scale, IBD DISK (adapted), and PHQ-2/GAD-2.

**Conclusions:**

The identified treatment targets differed from those prioritized in clinical trials. However, DGBI and IBD literature suggests that many of the identified constructs supersede traditional outcomes such as anxiety and depression. These results can guide integration of patient-reported outcome measures into gastropsychology programs and highlight the value of involving patients in program development.

**Supplementary Information:**

The online version contains supplementary material available at https://doi.org/10.1186/s41687-026-01125-x.

Chronic digestive disorders are common, costly, and have significant negative impacts on quality of life and functioning [1–3]. The complex communication system between the brain and the gut (otherwise known as the brain-gut axis) is increasingly implicated in symptom severity, disease course, and disease burden, across gastrointestinal (GI) conditions. Additionally, digestive disorders are associated with increased risk of mental health conditions including, but not limited to, anxiety, depression, and insomnia [4–6]. Understanding the bidirectional relationship between GI and mental health symptoms is critical to providing high quality care for this population. At present, integration of behavioral health within gastroenterology is considered best practice and is linked to a number of positive outcomes including increased patient satisfaction, decreased provider burden, and improved symptom burden [7–12].

A broad range of evidence-based brain-gut behavior therapies are available for treatment of disorders of gut-brain interaction (DGBI) and inflammatory bowel disease (IBD) [13], often termed *brain-gut behavior therapies*. Although there is no clear evidence that one particular type of therapy is more effective than another, cognitive behavior therapy (CBT) and gut directed hypnotherapy (GDH) have the most research supporting their effectiveness and are most widely implemented. Notably, treatment studies so far have largely focused on primary outcomes of physical and mental health symptoms. Two notable randomized controlled trials have found CBT for IBS to be effective in decreasing symptom severity compared to control conditions, with effects maintaining at 6–12 month follow ups [11, 14]. CBT has also been shown to decrease symptoms in upper DGBI conditions [15–17]. In addition to measuring symptom reduction, mental health outcomes, most often anxiety, depression, and quality of life, are shown to improve with CBT for DGBI and IBD [18–22]. Additionally, there is some evidence to support the reduction in IBS symptoms independent of psychological distress which indicates direct impact on the gut-brain axis [23]. Regarding GDH, the most recent review and meta-analysis of GDH for IBS found treatment to be superior to comparative conditions for improvements in global IBS symptoms and pain [24]. Clinical trials of GDH in other DGBI and IBD also prioritize symptom reduction or maintenance of remission as the primary outcome of interest and show promising results [25–28].

While results from these trials are integral to improving access to high quality, evidence-based care, less is known about the role of brain-gut behavior therapies on factors prioritized by patients who are living with GI conditions. Further, clinical trials examining efficacy of an intervention can differ significantly from clinical practice [29]. Measures used in clinical trials are often extensive and research coordinators track their completion through frequent prompts and scheduled follow-up assessments, a process which may not be translatable to a clinical context. Additionally, the extent to which interventions are deemed effective depends, in part, on the manner in which change is measured. The use of patient-reported outcomes measures (PROMs) in behavioral interventions has demonstrated benefit and is viewed favorably by providers, though actual use lags behind favorability [30, 31]. The use of PROMs emerged as a strategy for measuring change in health outcomes with the recognition that change in an objective (e.g., physical or organic) variable does not necessarily equal change in function, symptoms, or quality of life in patients [32]. Additionally, the established importance of assessing patient preferences to improve healthcare outcomes (e.g., shared decision-making) adds further urgency to engaging patients in the selection of outcomes measurement [33, 34]. The thoughtful selection of patient-centered outcomes is thus essential to ensuring that measures represent the domains that are most central to improvement in patients and that interventions are designed and delivered accordingly. Engaging patients as stakeholder participants to guide measurement selection is a method that can increase the likelihood that the measures used to assess change map onto the domains most important to patients [35]. Thus, the aim was to determine the optimal PROMs to assess change and improvement following participation in brain-gut behavior therapies using a stakeholder-engaged process combining individual qualitative interview and focus group data.

## Methods

### Stakeholder engagement

Stakeholder engagement is defined as the “iterative process of actively soliciting the knowledge, experience, judgment and values of individuals selected to represent a broad range of direct interest in a particular issue, for the dual purposes of: creating a shared understanding; making relevant, transparent and effective decisions [36].” Stakeholder engagement is a spectrum of involvement in the research and quality improvement enterprises ranging from setting research priorities and guiding clinical trial design in the earliest phases to implementation and evaluation at the later stages [37, 38] Although stakeholder engagement can occur anywhere along this spectrum, the involvement of patients tends to occur most frequently during evidence generation at the level of human clinical trials through participation in trial design and methods (e.g., patient recruitment strategies) [39].

### Design and population

We used a rapid qualitative analysis (RQA) approach to understanding and characterizing patient preferences for prioritizing PROMs [40, 41]. We first conducted individual qualitative interviews with patients who represented a cross-section of two dimensions: (1) those with organic conditions or DGBI and (2) those who did and did not have prior experience receiving treatment from an embedded GI behavioral health program. We subsequently conducted focus groups with different patients to gain participant feedback on prioritization of possible domains of assessment identified during qualitative interviews. All interviews and focus groups were conducted via the Webex videoconferencing platform, recorded, and automatically transcribed by the Webex integrated transcription software.

We recruited patients receiving services in the Center for Digestive Health within the Section of Gastroenterology and Hepatology at Dartmouth Hitchcock Medical Center through outreach to gastroenterologists, advanced practice providers, and behavioral health providers. We also conducted chart reviews to identify additional patients to fill underrepresented sample cells, primarily to address an overrepresentation of patients with DGBI who had already participated in behavioral health interventions. Patients in those groups (e.g., DGBI without behavioral health intervention; organic condition with behavioral health intervention) were also contacted by their GI providers and invited to participate. Interview participants were compensated $50 for their time. Focus group participants were also contacted by providers and asked to participate; participants were scheduled such that each focus group had a mix of participants with organic GI conditions and DGBI and with mixed experiences with GI behavioral health treatments. Focus group participants were not compensated for their time. This study received approval from the Dartmouth Health Human Research Protection Program (STUDY02002211) and participants provided their informed consent prior to engaging in any study activities.

#### Qualitative interviews

An initial draft of a semi-structured qualitative interview was developed by REB and JSD focusing on lived experience of disease burden, treatment outcomes and goals, perceived effects of behavioral health interventions, treatment components, recommendations to gastrointestinal care providers, and outcomes to assess, with the goal of balancing interview duration with depth of questions. The interview guide was piloted with two patients who had experience with the GI behavioral health program and were able to provide feedback on the adequacy of the interview to achieve the project goals. The interview was subsequently revised based on this feedback for content and duration prior to data collection. We selected RQA as our primary approach on an a priori basis which also partly informed the decision-making process as we considered interview structure and content. One of the core features of RQA is the premium placed on brevity and conciseness over depth of resulting data. Therefore, the duration of the resulting interview was intentionally limited to approximately 30 min.

The interviews were structured with nine core questions asked to all patients with sub-questions and prompts as needed. An additional set of questions were asked of patients on the basis of their history of engagement with the behavioral health services. Patients who were currently or previously engaged with behavioral health were asked an additional six questions focused on their experiences with those services. Patients who had not previously engaged with behavioral health services were asked four additional questions assessing the perceived reasons that they were or were not referred, interest in services, barriers to engagement, and what providers should know about coping with GI conditions. Finally, patients were also asked directly what they perceived to be the most important outcomes to measure during GI behavioral health treatment. The semi-structured interview guides are included as a Supplemental File. All interviews were conducted by the first author (REB) who is a clinical psychologist with expertise in outcomes evaluation working outside of the gastrointestinal medicine field and without lived experience with chronic GI illness.

#### Rapid qualitative analysis (qualitative interviews)

We used the Planning for and Assessing Rigor in Rapid Qualitative Analysis (PARRQA) to develop and conduct this project [42]. The PARRQA framework is presented as a checklist in the Supplemental file. Consistent with the guidelines of published reports on the use of RQA and the PARRQA framework, we developed and pilot tested a summary template. The summary templates were constructed to provide a concise document for efficiently organizing interview data. All questions were associated with a domain name, with 1–2 questions per domain (e.g., “What would be different in your life if [GI condition] was suddenly cured?” related to Idealized Outcomes domain). The analytic team consisting of REB and JSD independently summarized two interview transcripts and compared summaries to ensure that the summary templates adequately captured the relevant patient response content. Subsequent interview summaries were split roughly equally between these two study personnel for summarization.

The data from the summaries were then transferred to a summary matrix using Excel designed such that the data from the summaries were organized with patients as rows and domains as columns, allowing for a quick review of the qualitative data by domain. The analytic team then independently conducted an initial synthesis of the domains, resulting in memos summarizing the data for each domain across cases. Finally, the team met again to combine and review the consolidated memos and extract themes from the data. These themes formed the overarching topic introductions for the subsequent focus groups as well as initial considerations of the optimal PROMs to be further deliberated during the focus groups.

#### Focus groups

Our approach to focus group composition, size, and content focus was guided by best practices in qualitative data collection and intended to facilitate discussion among patients with DGBI who could advise on the optimal outcomes based on the results from our RQA [43]. Three sixty-minute focus groups consisting of 3–6 patients were led by CAS; focus group participants had not participated in qualitative interviews. Discussion in each focus group followed a semi-structured guide, as follows: first, focus group participants were provided with a brief overview of the field of gastropsychology and the GI Behavioral Health program at DHMC. Participants were then oriented to the overall goal to “determine what we should measure before and after GI behavioral health treatment to understand if treatment helped.” Additional goals were to “gain patient feedback on the common ideas from our interviews” and “take patient perspectives and develop a set of questions we can ask patients regularly during treatment.” Possible outcomes to measure (derived from interview content) were shared with participants, following which they were asked which outcomes they thought were most important and if they thought any outcomes were missing. Following discussion and prioritization of themes, participants were encouraged to discuss how long a PROM battery should take, how often PROMs should be assessed, and how they wanted feedback on their responses.

#### Focus group data analysis

Focus groups were audio recorded and transcribed using the Webex videoconferencing platform and embedded software. The analytic team reviewed the transcripts of the focus groups, individually recorded memos of reactions to those groups in line with each of the specific prompts posed to participants, and then collaboratively summarized overarching perspectives.

**Fig. 1:**
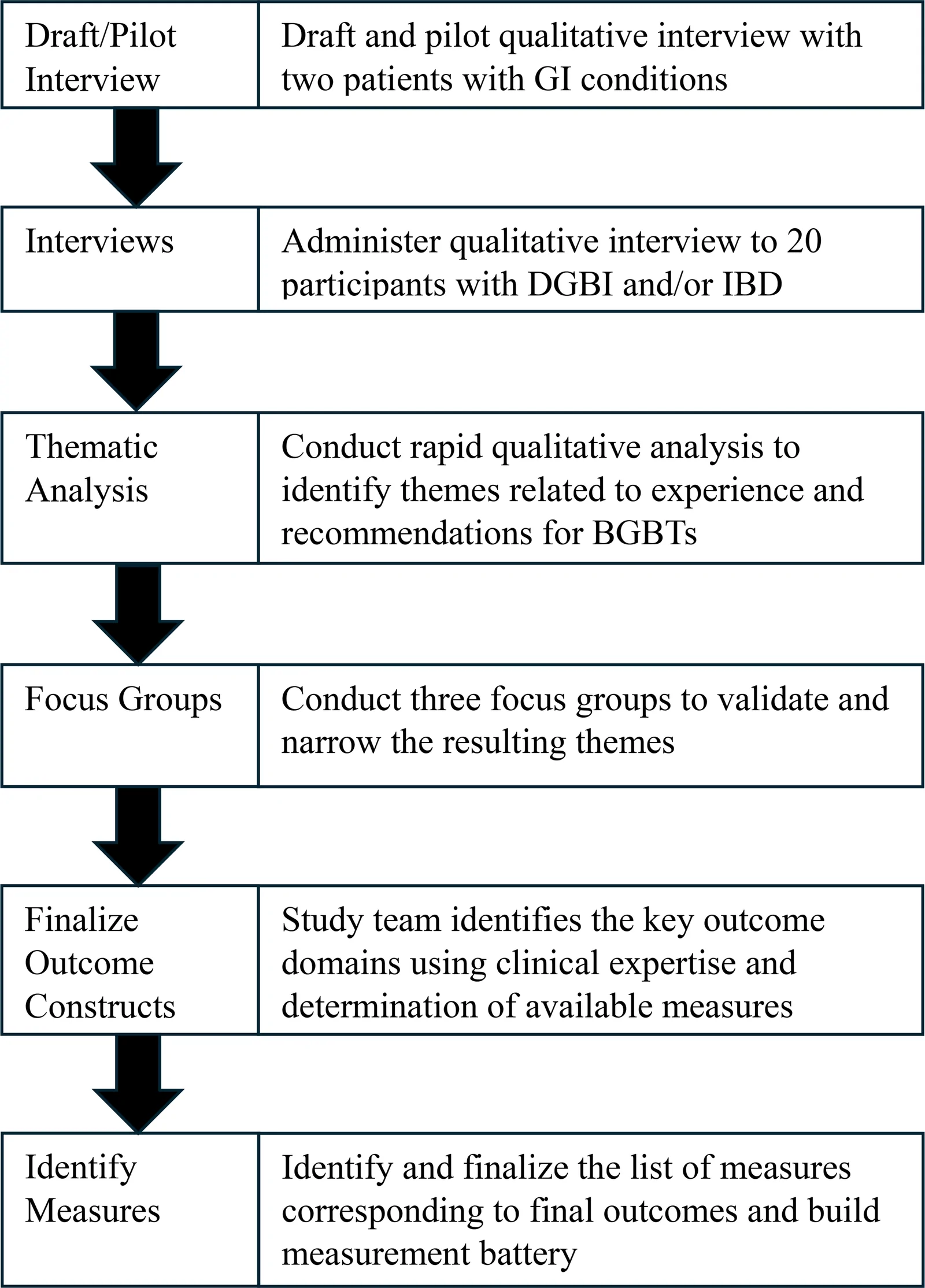
Study process diagram. Notes. DGBI, disorders of gut-brain interaction; IBD, inflammatory bowel disease; BGBT, brain-gut behavior therapies

Figure 1 includes an overview of the core study processes.

## Results

Qualitative interview participants included 20 patients with one or more GI conditions. Fourteen participants had a diagnosis with an identifiable organic cause (e.g., Crohn’s disease), with the remaining six having a DGBI (e.g., irritable bowel syndrome). Of the 14 participants with an organic GI condition, four had completed one or more encounters with a behavioral health clinician through the gastropsychology clinic housed within the outpatient GI clinical service. Five of the six participants with a DGBI had completed one or more visits with a behavioral health clinician. 60% of the sample was female and one participant endorsed a non-binary gender identity. Additional demographic features of the sample are depicted in Table 1.

**Table 1:** Aggregated participant demographics for qualitative interview sample (*N* = 20)

	Overall Sample	GI-BH Patients (*n* = 9)	Non-GI-BH Patients (*n* = 11)
Age	Range: 23–81 Mean = 49.45 SD = 17.84	Range: 23–79 Mean: 44.44 SD: 17.95	Range: 29–81 Mean: 53.55 SD: 17.49
Gender	Women: 11 (55%) Men: 7 (35%) Other: 2 (10%)	Women: 4 Men: 3 Other: 2	Women: 7 Men: 4 Other: 0
Race	White: 17 (85%) American Indian/Alaska Native: 2 (10%) Black or African American: 1 (5%)	White: 7 American Indian/Alaska Native: 1 Black or African American: 1	White: 10 American Indian/Alaska Native: 1 Black or African American: 0
Ethnicity	Non-Hispanic: 19 (95%) Hispanic or Latino: 1 (5%)	Non-Hispanic: 9 Hispanic or Latino: 0	Non-Hispanic: 10 Hispanic or Latino: 1

Note: significance tests not available for several demographic variables due to low Ns in categories

Focus groups included 14 additional patients with a similar mix of IBD and DGBI and with and without past GI behavioral health experience. Focus group participants also varied in age and gender, similar to the qualitative interview sample. Focus group participation was anonymized (with the exception of identification during the groups themselves) and thus specific demographics on this subsample are not available for inclusion.

### Qualitative interviews

Our inductive analysis yielded 11 themes corresponding to domains assessed in the interviews. These were reduced from the original planned 15 domains assessed in the interviews. The removed domains and those consolidated into other domains were *Unimportant Outcomes* (removed due to minimal responses), *Perceptions of Referral* and *Reasons for Non-Referral* (consolidated into *Reasons for Referral/Non-Referral*), and *Reasons to Attend Behavioral Health* and *Reasons to Not Attend Behavioral Health* (consolidated into *Reasons to Attend/Not Attend Behavioral Health*). We then further consolidated the original domains into five concise domains: *Lived Experience of Disease*, *How to Cope with Illness*, *Perceived Value of Behavioral Intervention*, *Outcomes and Measurement*, and *Behavioral Intervention Effects*. These domains, corresponding interview items, resulting themes, and representative quotations are summarized in Table 2.

**Table 2:** Qualitative domains, interview items, themes, and representative quotations

Domain	Interview Items	Theme	Representative Quotations
Lived Experience of Disease	What is it like living with [GI condition]?	GI conditions are unpredictable, uncertain, and require intensive planning.	“A constant fear of the unknown and trying to plan for the future.” (ID 21)
	What is the worst part of living with your GI condition?		“There’s just like, not really a whole lot of predictability and when my symptoms are going to act up. So that, I think is the piece that affects everything” (ID 15)
How to Cope with Illness	What advice would you give to someone else trying to manage [GI condition]?	Flexibility, acceptance, and experimentation are important components of coping (Coping Strategies)	“Find time to lose time and anticipate rest… it’s important to know that if plans need to change, you should give them the grace to change and work with your body instead of against it.” (ID 1)
	What skills, tools, or techniques do you use that you learned in your GI BH treatment? (GI BH Patients)	Multiple skills were useful, rather than one specific strategy (Acquired Skills)	“It made me more aware that I do have some control, that there’s practices I can do that will make me be able to manage my life.” (ID 14)
Perceived Value of GI BH	Have you ever been referred to GI BH treatment? If yes: What did you think about the referral? If no: Why do you think that is?	Services were unnecessary for people with low symptom burden (Reasons for Referral/Non-Referral)	“[Gastroenterologist] has always answered all my questions so I never really felt puzzled or left wanting more help, but I just never thought of it and I think it would be helpful.” (ID 20)
	Would you be interested in receiving GI BH treatment for your [GI condition] in the future? If yes: What do you hope would change as a result of working with GI BH?	Services may be most useful during a GI disease flare and access barriers were largely practical (Reasons to Attend/Not Attend)	“It would be nice to be able to have a team of psychologists who specialize in your specific medical illness.” (ID 21)
	Are there any reasons you wouldn’t want to do a GI BH intervention?		“I don’t feel pressed by this disease right now.” (ID 10)
	Are there any downsides to GI BH treatments that they should be aware of?	It is important to set expectations for treatment effects, but that otherwise downsides are minimal (Negatives of Treatment)	“I was kind of hoping that we were gonna take things a step deeper and we just didn’t do that.” (ID 15)
Outcomes and Measurement	What would be different in your life if [GI condition] was suddenly cured?	People wish to re-engage in meaningful areas of their life and almost nothing goes unaffected (Important Outcomes)	“I would feel freer to do things like travel and, you know, I just wouldn’t feel as, maybe hesitant at times.” (ID 20)
	What are some of your hopes about the future with regard to how [GI condition] is managed?		“To just be able to live a life that’s not centered around my bowels.” (ID 15)
	What do you think we should be measuring?	GI BH treatment should assess anxiety, depression, activity engagement, quality of life, and self-efficacy for managing chronic disease (GI BH Measurement).	“How it impacts your day-to-day life. How that range has increased or decreased with therapy” (ID 24)
GI BH Intervention Effects	How did your GI BH health treatment help your GI symptoms?	GI BH increased perceived ability to cope more than reduced physical symptoms (GI BH Effects on Physical Systems)	“Because you have tools to deal with the problem, you don’t feel helpless” (ID 14)
	How about coping with the condition or other parts of life?	GI BH increased social connectedness, symptom communication, and some psychological symptoms (GI BH Effects on Non-Physical Systems)	“I was having a difficult time mentally handling that (flare) and I know I was able to turn things around…staying on top of how I was feeling mentally helped.” (ID 1)
	Besides the GI symptoms, what areas of life were improved by your GI BH treatment?		“I’m able to verbally explain, calmly explain how I’m feeling as opposed to something sets me off that has nothing to do with why I’m upset. Communication is definitely a lot better.” (ID 24)

Abbreviations: GI, gastrointestinal; BH, Behavioral Health

*Lived Experience of Disease* reflects the insights that participants provided on what it is like to live with a GI disease. The single theme within this domain was that GI conditions are unpredictable, uncertain, and require intensive planning. All participants described a perception that it was difficult or impossible to predict when a flare or specific symptom would impact them, and that as a result of this unpredictability they would seek to prepare for the worst. Uncertainty was a common experience across participants.

*How to Cope with Illness* comprises coping strategies and acquired skills. Coping strategies were the ways that participants managed their illness prior to receiving any behavioral health interventions or continued to use in the absence of additional services. In other words, these were the approaches that patients innately used or had learned from their own experience. In contrast, acquired skills were the specific techniques learned in the course of engaging in behavioral health interventions. The themes derived for this domain are: (1) Flexibility, acceptance, and experimentation are important components of coping (Coping Strategies) and (2) Multiple skills are useful, rather than one specific strategy (Acquired Skills).

*Perceived Value of Behavioral Intervention* included participant insights on the perceived reasons for being referred or not referred for behavioral health interventions, reasons to attend or not attend those interventions, and potential negatives or downsides of those interventions. The corresponding themes were: (1) Services were unnecessary for people with low symptom burden (Reasons for Referral/Non-Referral), (2) Services may be most useful during a GI disease flare and access barriers were largely practical (Reasons to Attend/Not Attend), and (3) It is important to set expectations for treatment effects, but that otherwise downsides are minimal (Negatives of Treatment). Overall, there was a sense among participants that the value of these interventions was tied to the severity and impairment from illness at any given time, but that there were minimal negatives to these interventions.

*Outcomes and Measurement* combines participants’ opinions on the most important and desirable outcomes of behavioral health interventions and how best to measure these outcomes. Themes within this domain include: (1) People wish to re-engage in meaningful areas of their life and almost nothing goes unaffected (Important Outcomes) and (2) GI behavioral health treatment should assess anxiety, depression, activity engagement, quality of life, and self-efficacy for managing chronic disease (Behavioral Health Measurement). Although participants identified several symptom or life domains that were important to measure, there was a general sense that these domains largely related to a return to engagement in usual life events and activities.

Finally, *Behavioral Intervention Effects* include the perceived behavioral health intervention effects on physical and non-physical (e.g., emotional) systems. The corresponding themes were: (1) Behavioral treatments increased perceived ability to cope more than reduced physical symptoms (Intervention Effects on Physical Systems) and (2) Behavioral health interventions increase social connectedness, symptom communication, and some psychological symptoms (Intervention Effects on Non-Physical Systems). Thus, while there were some recognized effects on physical symptoms, the predominant benefit of treatment was on the non-physical experience of GI illness.

### Focus groups

During subsequent focus groups, participants broadly highlighted the importance of multiple constructs derived from qualitative interviews. Primarily, participants across the three focus groups prioritized gaining confidence in symptom management, enabling them to be more engaged in their daily life. For example, one participant suggested asking, “Are you making progress in being part of the world?” as a key area of assessment. Relatedly, participants shared how increased ability to participate in daily life would support social connectedness. Several of those who had participated in behavioral health groups also shared how the group experience supported social connectedness and reduced experiences of isolation. Across focus groups, participants highlighted the importance of measuring some aspect of isolation/connectedness. With regard to symptom experiences, participants prioritized assessment of lived experiences with these diseases, including “thoughts and feelings” about having and managing a GI condition. Several participants noted anxiety and fear as particularly important in this process, or as one participant shared, “how much do you feel like you’re waiting for the other shoe to drop?” Finally, participants also named a number of symptom domains to be assessed, including, but not limited to, depression, anxiety, sleep, and pain.

With regard to the experience of completing questionnaires, participants recognized the value of regular, structured assessments as part of their care. While there was some variability in preferences for the length and frequency of the assessment battery, the majority of participants suggested that around 10 min would be the maximum length for their comfort. Participants also suggested administering the battery once per month or less. Importantly, many participants also indicated that for an initial visit, a questionnaire battery of a longer duration could be acceptable. Finally, participants broadly highlighted the importance of being able to view and engage with their own data as a way to collaborate on their own care.

### Measurement decisions

Based on the themes derived from the qualitative interviews, the authorship team developed a list of candidate measures to be used in a specialty GI behavioral health practice. Candidate measures were selected from the literature based on their length, availability, and prevalence in the literature. We only considered measures that had demonstrated multiple forms of validity and reliability across more than one validation study in one or more chronic illness populations. Following the collection of focus group data, we further narrowed this list to (a) prioritize areas highlighted by participants and (b) take less than 10 min of total battery administration time. Based on published methodology for determining survey length [44, 45], we aimed for a maximum of 30 items in our clinic battery. Final questionnaires included in our assessment battery are as follows:

- *Brief Illness Perception Questionnaire* [46], a 9-item questionnaire designed to understand the cognitive and emotional experiences of a chronic illness.
- *PROMIS Self-Efficacy for Managing Chronic Conditions – Symptoms* [47], a 4-item questionnaires that assesses confidence in management of symptoms across domains (e.g., in relationships, in public, collaboration with healthcare providers).
- *UCLA Loneliness Scale* [48], a 3-item questionnaire that assesses experiences of connectedness and isolation.
- *IBD DISK (adapted)* [49], a 10-item questionnaire that was designed to assess IBD-related disability, or the ways in which IBD affects functional status across 10 domains. Language was adapted herein to reference “GI illness” as opposed to “IBD” in questionnaire language and instruction.
- *PHQ-2/GAD-2* [50], a 4-item questionnaire designed to screen for presence of depression and anxiety.

A list of candidate questionnaires, final questionnaires, and how they map on to interview-derived themes is included in Table 3. In our own clinical practice, this battery of questionnaires is assigned to every new patient visit and the first visit for every therapy group. The battery is automatically sent to patients 6 weeks after it was assigned, though if they have filled out any of the questionnaires within the last 2 weeks (e.g., because they started another therapy group or filled out the same measure in another department), the questionnaire will not repeat.

**Table 3:** Integration of qualitative domains with validated questionnaires, including rationale for final decisions

Domain	Candidate Questionnaires	Final Questionnaire	Rationale
Lived experience of disease	Brief Illness Perception Questionnaire Fear of Uncertainty Scale Connor-Davidson Resilience Scale	Brief Illness Perception Questionnaire	Addresses fear related to having a chronic disease, in addition to overarching cognitive and emotional experiences
How to cope with illness	Coping Flexibility Questionnaire Connor-Davidson Resilience Scale Visceral Sensitivity Index	Brief Illness Perception Questionnaire	Addresses cognitive and emotional factors related to the illness experience.
Outcomes and Measurement	PHQ-9 GAD-7 Insomnia severity index IBS Severity Scale Pain Catastrophizing Scale IBD-DISK	IBD-DISK PHQ-2 GAD-2	IBD-DISK (adapted to be generally about one’s GI illness) quickly assesses a number of distinct domains, but does not specific anxious versus depressive symptoms, thus PHQ-2 and GAD-2 were added.
Behavioral intervention effects	Brief Illness Perception Questionnaire PROMIS Self-Efficacy for Managing Chronic Conditions UCLA loneliness scale	PROMIS Self-Efficacy for Managing Chronic Conditions UCLA loneliness scale	Broadly addresses confidence in managing a disease, with specific items that address a number of common concerns such as going out in public and communicating successfully with one’s medical team. Measure on loneliness addresses a key concept highlighted in both study phases.

Note: questionnaires were not assigned to the Perceived Value of Intervention domain, as this theme broadly highlighted the importance of timely care (i.e., during symptom exacerbations) as opposed to an explicit construct to be measured

## Discussion

People with GI conditions report varied symptom experiences, and mental health comorbidities are common. In clinical trials of brain-gut behavior therapies, including those focused on IBD and DGBI, primary and secondary outcomes typically include GI symptoms (e.g., abdominal pain, bowel frequency), quality of life, and overarching mental health symptoms (e.g., anxiety, depression). [18, 22, 24, 27] While these targets are important and modifiable according to trial outcome data, it is not known whether they are consistent with *patients’* desired areas for intervention. Consistent with the recognized importance of applying patient preferences to intervention development, refinement, and implementation (e.g., shared decision making), we sought to identify the constructs that patients believed were most important for a GI behavioral health program to measure to determine changes in their disease experience and treatment outcomes.

Through qualitative interviews with a participant sample representative of our clinic population, we identified 5 core domains that encapsulate disease management through a GI behavioral health lens: *Lived Experience of Disease*, *How to Cope with Illness*, *Perceived Value of Behavioral Intervention*, *Outcomes and Measurement*, and *Behavioral Intervention Effects*. The domains were further composed of 10 themes that represented core constructs in the management of disease, including behavioral health strategies and effects of interventions. Constructs derived from these themes were then presented to a similar participant population in a series of focus groups to further refine our assessment targets. Broadly, focus group participants reinforced information derived from qualitative interviews highlighting the importance of assessing an intervention’s effectiveness at helping them to reengage in important areas of life. Indeed, an overarching theme was the value placed in getting them back to a “normal” life through the use of a flexible armamentarium of coping skills regardless of the degree to which they alleviated physical symptoms. The resulting selection of measures aims to do this by focusing on perceptions of disability, ability to cope, social connectedness, and emotional distress.

A multitude of disease management skills are either already possessed by individuals living with GI conditions or acquired through participation in behavioral health interventions in the GI specialty setting, according to participants in this study. Although there are a variety of the skills endorsed by this sample as useful for coping with these conditions, there was a general impression that learning to live flexibly through acceptance of symptoms in the moment while also experimenting with different techniques was key to regaining a sense of normalcy. Accordingly, there was no one skill or technique that was responsible for the greatest degree of success in coping with GI illness. The effects of these interventions were primarily on non-physical aspects of illness such as improved communication about symptoms and increased social connectedness, effects that would not be readily detected through physical examination or testing. In general, there was a sense of increased psychological relief in the form of decreased anxiety coupled with an increased sense of control and expectation that they could weather acute symptom flares.

The increased ability to cope with symptoms more than decreasing physical symptoms themselves was reflected in participants’ impressions of the value of behavioral health interventions for GI disease. These interventions were viewed as most relevant in the context of symptom flares and were less acutely needed for those participants with milder, well-controlled symptoms or in an interval of dormant illness. Despite the absence of need for intervention among those participants’, the behavioral health interventions were still viewed positively and without specific drawbacks aside from practical barriers (e.g., scheduling). The lone exception to this general positive impression was that expectations should be set appropriately so that patients do not engage in these interventions expecting complete alleviation of all physical symptoms.

Finally, participants identified measurable outcomes that are largely aligned with the perceived effects and value of behavioral health interventions targeting GI diseases, as well as the most challenging aspects of their lived experience. The overarching statement was that measurement of the degree to which intervention results in an ability to re-engage with the most meaningful aspects of daily life and associated activities of daily living is critical to assessing the success of behavioral health interventions. Although much of those associated impairments are a direct result of physical symptoms, a substantial proportion of impairment comes from the emotional and behavioral consequences of illness, such as anxious avoidance, depression, and low self-efficacy. The focus groups further solidified those impressions by endorsing the use of brief measures that assessed coping and activity engagement along with perceived ability to cope with illness. A final list of measures was developed following completion of interview and focus group data collection. The final list of measures included the *Brief Illness Perception Questionnaire* [46], *PROMIS Self-Efficacy for Managing Chronic Conditions – Symptoms* [47], *UCLA Loneliness Scale* [48], *IBD DISK (adapted)* [49],and *PHQ-2/GAD-2* [50].

While the subset of measures derived from these qualitative data are less commonly primary or secondary trial outcomes, many of the measures have been used in prior GI research. One recent study demonstrated that perceptions of illness were different in people with IBS and IBD; for example, people with IBS reported higher perceived consequences of their illness, but lower perceived understanding of their illness. [51] In both IBS and IBD, overall perceived threat of illness was associated with symptom severity. Two other studies in IBD have demonstrated that disease severity predicts illness perceptions, while illness perceptions impacted stress, anxiety, and depression, supporting the potential impact of targeting this key factor in disease experience. [52, 53] Research in IBS is similar, and has demonstrated that changes in illness-related cognitions may be more important than changes in mood in improving IBS-related outcomes. [54] Research on loneliness in both IBD and IBS is similar. A recent review on loneliness in IBD demonstrated that loneliness is associated with a number of consequences, including greater disease burden and disease activity and reduced resilience and quality of life. [55] Similarly, a large, longitudinal study found that loneliness and social isolation increased one’s risk for developing IBS, highlighting social connectedness as a potential intervention target. [56] Finally, while researchers use a variety of measures to assess self-efficacy, the literature broadly suggests that greater self-efficacy is associated with better physical and mental health in DGBI and IBD. [57–59] Thus, our research highlights that patients’ perspectives on important areas to target are consistent with cognitive and behavioral processes that are known to contribute to disease outcomes. Further, these patient-identified areas of interest may supersede commonly researched constructs like anxiety and depression.

This research is an important step toward giving patients a voice in their own care, but also has limitations. Importantly, the themes derived from this qualitative work are complex, and may not be fully represented by a brief questionnaire battery. Ideally, clinicians would have sufficient time and resources to assess the domains that are important to each patient based on their values and disease experience, and then could monitor those individualized constructs over time. However, that degree of individualization is impractical in the context of a high-volume treatment program. Additionally, electronic medical record systems can pose barriers to deployment of measures individualized to each patient’s specific valued outcomes. Future work is needed to determine whether this succinct, patient-focused assessment battery is sufficient for detecting changes within individuals and between therapy techniques. This work is currently underway, and will further our understanding of the limits of brief, static measurement strategies. Finally, while the participants were representative of the clinical setting in which this research occurred, future research is needed to understand whether the constructs identified herein are generalizable to other settings (i.e., more racially diverse or more urban environments). There may also be meaningful differences in outcomes based on other demographic features. For example, the average ages for our samples of patients with and without prior experience with BGBTs differed by 10 years. It is possible that perspectives on what constitutes an important outcome may vary not just by experience with BGBTs, but by age or other demographic variables that have not yet been examined.

## Conclusions

While much of the literature on brain-gut behavior therapies has prioritized GI and mental health symptoms as primary and secondary outcomes, these treatment targets may not be aligned with patients’ goals for care. In this two-phase qualitative study, we identified constructs that patients believe are important treatment targets for a GI behavioral health program, sought patient guidance on how to measure those constructs in clinical practice, and designed an assessment battery aligned with patient perspectives. Constructs such as engagement in daily life, illness perceptions, and confidence in managing one’s GI condition not only were prioritized by patients, but also coincide with research suggesting that these constructs supersede traditional outcomes such as anxiety and depression. While additional research is needed, the results presented herein highlight the importance of partnering with patients in all aspects of program development and sustainment.

## Supplementary Information

Below is the link to the electronic supplementary material.

Supplementary Material 1

## Data Availability

Datasets generated and/or analyzed during the current study are not publicly available due to the personal nature of qualitative data. A limited dataset may be made available upon reasonable request to the corresponding author.
