# Supplementary material for: Patient Identification of the most important outcomes in a gastrointestinal behavioral health program
Source: J Patient Rep Outcomes. 2026 Jul 6;10:175. doi: 10.1186/s41687-026-01125-x (PMC13627641; doi:10.1186/s41687-026-01125-x)
Supplement: Supplementary file 1 [file 41687_2026_1125_MOESM1_ESM.docx]

| **Planning for and Assessing Rigor in Rapid Qualitative Analysis (PARRQA) Checklist** | | | |
| --- | --- | --- | --- |
| **Domain** | **Item No.** | **Guideline** | **Page No.** |
| Rigorous Design | 1 | Articulate the research question and finite purpose of the project | 5 |
|  | 2 | Describe the rationale for using rapid qualitative methods | 7-8 |
|  | 3 | Define what is meant by “rapid qualitative analysis” | 7-8 |
|  | 4 | Consider whether a theory, model, or framework will be used to inform the study and if so, why and how | * |
|  | 5 | Define the intended timeframe of data collection, analysis, and products/deliverables | * |
|  | 6 | Plan for appropriate staffing | * |
|  | 7 | Explain the purpose and timeline of the study, communication plan, and roles to all team members | * |
| Data Collection | 8 | Develop and refine data collection tools to address specified research questions | 6-9 |
|  | 9 | Pilot data collection instruments to ensure they are clear, feasible, and appropriately targeted | 6-7 |
|  | 10 | Develop a plan for review throughout the data collection process | * |
| RQA: Summary Template Development | 11 | Develop and pilot test a user-friendly summary template | 8 |
|  | 12 | Ensure there are cross-references to raw data to support continuous comparison and validation necessary for RQA | 8 |
|  | 13 | Develop summaries that are accurate and concise, but detailed enough to meet project aims | 8 |
|  | 14 | Identify training and calibration processes to ensure consistency and accuracy in summaries | 8 |
| RQA: Matrix Analysis | 15 | Plan qualitative matrix structure to reflect project aims/ questions | 8 |
|  | 16 | Describe use of software for matrix analysis (e.g., Excel, Word) | 8 |
|  | 17 | Develop a plan for review throughout matrix analysis | 8 |
| Rapid Qualitative Data Synthesis | 18 | Conduct synthesis that is rigorous and responsive to research priorities | 10-12 |
| Note. The Planning for and Assessing Rigor in Rapid Qualitative Analysis (PARRQA) framework includes items intended for use in planning and design, rather than reporting. Items specific to the planning phase are denoted with an asterisk (*). | | | |
